# Repeated Paced Mating Increases the Survival of New Neurons in the Accessory Olfactory Bulb

**DOI:** 10.3389/fnins.2020.00249

**Published:** 2020-03-24

**Authors:** Wendy Portillo, Georgina Ortiz, Raúl G. Paredes

**Affiliations:** ^1^Instituto de Neurobiología, Universidad Nacional Autónoma de México (UNAM), sQuerétaro, Mexico; ^2^Escuela Nacional de Estudios Superiores, Unidad Juriquilla Universidad Nacional Autónoma de México (UNAM), Querétaro, Mexico

**Keywords:** paced mating, cell survival, neuronal differentiation, sexual experience, olfactory bulb

## Abstract

In female rats, the first sexual experience under paced mating conditions increases the number of newborn cells that migrate into the granular layer of the accessory olfactory bulb (AOB). Repeated paced mating has a potentiating effect on the number of new neurons that migrate to the AOB compared with a single session 15 days after paced mating. On the other hand, one paced mating session does no increases the survival of new cells 45 days after mating. In the present study, we evaluated if four paced mating sessions could increase the survival of new neurons in the AOB and main olfactory bulb (MOB) 45 days after females mated. Sexually naive female rats were ovariectomized, hormonally supplemented and randomly assigned to one of five groups: (1) Control, no sexual contact (C); (2) Four sessions in which females were exposed, without mating, to a sexually experience male rat (SE); (3) One session of paced mating (PM1); (4) Four sessions of paced mating (PM4); and (5) Four sessions of non-paced mating (NPM4). In the first behavioral test, females received the DNA synthesis marker 5-bromo-2′deoxyuridine and were euthanized 45 days later. Our data showed that the number of new cells that survived in the mitral cell layer of the AOB decreased when females were exposed to a sexually active male, in comparison to females that mated once pacing the sexual interaction. Repeated sexual behavior in pacing conditions did not increase the survival of new cells in other layers of the MOB and AOB. However, a significant increase in the percentage of new neurons in the granular and glomerular layers of the AOB and granular layer of the MOB was observed in females that mated in four sessions pacing the sexual interaction. In the group that paced the sexual interaction for one session, a significant increase in the percentage of neurons was observed in the glomerular layer of the AOB. Our data suggest that repeated paced mating increases the percentage of new neurons that survive in the olfactory bulb of female rats.

## Introduction

Mating is a rewarding behavior that induces physiological and plastic changes. Female rats in natural, semi-natural and laboratory conditions can pace the sexual interaction, controlling the frequency and intensity of the sexual stimulation they receive (McClintock and Adler, 1978; Erskine, 1989). Mating in pacing conditions induces a reward state evaluated by the conditional place preference (CPP) test in male and female rats, mice and voles (Agmo and Berenfeld, 1990; Martinez and Paredes, 2001; Kudwa et al., 2005; Coria-Avila et al., 2006, 2008; Parada et al., 2012; Pfaus et al., 2012; Ulloa et al., 2018). When females or males mate without pacing the sexual interaction, this behavior does not induces a reward state (Martinez and Paredes, 2001; Ulloa et al., 2018).

Paced mating also induces plastic changes in the central nervous system. Adult neurogenesis is one of the most studied plastic changes. This process has been linked to reproduction from the early descriptions of its occurrence in adult life. Olfactory bulb (OB) neurogenesis in adult rodents is divided into three stages: proliferation, migration and survival. During proliferation (2 days) the stem cells replicate in the subventricular zone (SVZ) and rostral migratory stream (RMS). The new cells migrate (15 days) through the RMS and reach the glomerular (Gl), mitral (Mi), and granular (Gr) layers of the main and accessory olfactory bulb (MOB and AOB, respectively). The new cells that survive and integrate (45 days) into the OB layers become functional (Petreanu and Alvarez-Buylla, 2002; Winner et al., 2002). These stages are modulated by several internal and external factors. Thus, half of the new cells will die between 16 and 45 days after birth if they do not receive appropriate stimulation (Petreanu and Alvarez-Buylla, 2002; Winner et al., 2002).

The modulation of neurogenesis in the olfactory system in the adult brain in response to social and reproductive stimuli is well established (Peretto and Paredes, 2014; Bedos et al., 2018; Portillo et al., 2018). The first sexual experience in paced and non-paced conditions in female rats increases the proliferation of new cells in the RMS but does not increase the percentage of immature neurons (Corona et al., 2016). Paced mating increases the number of new cells that migrate to the Gr layer of the AOB but does not modify the number of new cells that survive in the MOB or AOB (Corona et al., 2011, 2016; Alvarado-Martinez and Paredes, 2018). However, the first paced mating experience increases the percentage of new cells that survive and differentiate into mature neurons in the Gr layer of the AOB (Corona et al., 2016). Constant sexual activity potentiates neurogenesis. Female rats that mate once a week for 4 weeks, pacing the sexual interaction, show an increase in the number of new cells and new mature neurons in the Gr layer of the AOB and MOB (16 days after their first sexual encounter) in comparison to females that mate four times under non-pacing conditions and females that mate once under pacing conditions (Arzate et al., 2013). Thus, repeated paced mating promotes the arrival of more cells that differentiate into mature neurons in the OB. In the present study, we evaluated if four sexual behavior tests before the critical time for cell survival (16 days) increases the number of new cells and neurons that integrate into the OB in female rats 45 days later.

## Materials and Methods

### Animals

**FIGURE 1:**
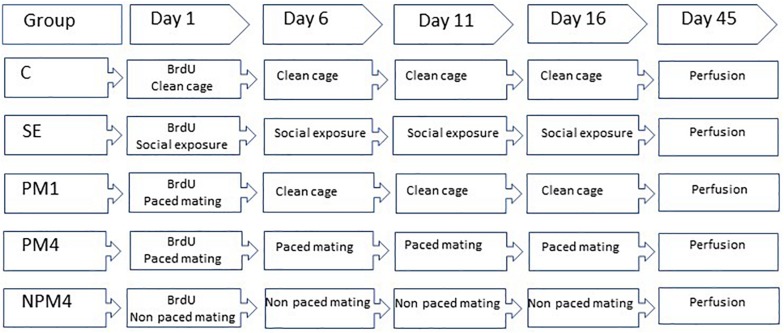
Schematic representation of the experimental groups and BrdU treatment. Sexually naïve female rats were ovariectomized and hormonally supplemented with estradiol and progesterone to induce sexual receptivity. Rats were randomly assigned to one of the following groups. Control (C), females that did not receive any sexual stimulation; Social exposure (SE), females exposed in four sessions to a sexually active male without mating; Paced mating one session (PM1), females mated once in pacing conditions; Paced mating four sessions (PM4), females mated in four sessions pacing the sexual interaction; and Non-paced mating four sessions (NPM4), females mated in four sessions without pacing the sexual interaction. BrdU was administrated 1 h before, immediately after and 1 h after the first mating test. Females were sacrificed 45 days after the BrdU injection.

Sexually naïve Wistar female rats (200–250 g), were obtained from a local colony at the Instituto de Neurobiología Universidad Nacional Autónoma de México. They were bilaterally ovariectomized and left 2 weeks to recover from surgery. As a stimulus, male rats (300–350) from the same strain were obtained and given sexual experience. They mated in three independent behavioral tests with receptive females that did not participate in the study. Only those males that ejaculated during the first 30 min of each test were included in the experiment. Females were randomly assigned to one of the following groups: (1) Control (C), females that did not receive any sexual stimulation; (2) Social exposure (SE), females exposed in four sessions to a sexually active male without mating; (3) Paced mating one session (PM1), females mated once in pacing conditions; (4) Paced mating four sessions (PM4), females mated in four sessions pacing the sexual interaction; and (5) Non-paced mating four sessions (NPM4), females mated in four sessions without pacing the sexual interaction. All groups had eight subjects except the SE group which had ten subjects. After the behavioral tests, all females returned to their home cage with rats of the same experimental group. To induce sexual receptivity all females were treated with estradiol benzoate (25 μg/kg) and progesterone (1 mg/kg) 48 and 4 h, respectively, before each behavioral test (Figure 1).

The experiments were approved by the Bioethics Committee of the Instituto de Neurobiología and were carried out in accordance with the “Reglamento de la Ley General de Salud en Materia de Investigación para la Salud” of the Mexican Health Ministry, which follows NIH guidelines.

### Behavioral Test

Behavioral tests were performed 5 days apart and lasted 1 h. Females mated or were exposed to their same male partner to receive similar sexual stimulation and avoid exposure to unknown male pheromones. Females from the C group were gently placed in a clean acrylic cage (40 × 60 × 40 cm) covered with fresh sawdust. SE females were placed in an acrylic cage equally divided into two compartments by an acrylic mesh with small holes that allowed visual, olfactory and auditory contact without physical interaction. The female was placed in one compartment and a sexually active male was placed in the opposite compartment. For the PM1 and PM4 groups, the acrylic cage was divided in two equal compartments by a removable screen. The female was placed in one side of the cage and the sexually active male was placed in the other side. The bottom of the screen had a hole in the middle big enough for the female, but not the male, to move freely from one compartment to the other. In this condition, females can move to the male compartment, mate and escape, pacing the sexual interaction. NPM4 tests were performed in the same acrylic cage without the screen, and a male was placed in the cage with the female. In this condition the male controls (paced) the sexual interaction since the female is not able to escape. The following parameters were recorded during the sexual behavior tests: number and latency of mounts, intromissions, and ejaculations; inter intromission interval obtained by dividing the ejaculation latency by the number of intromissions. For the female rats we registered the lordosis intensity (dorsiflexion posture that allows the penetration of the penis into the vagina) rated as “0” absence of the lordosis, “1” moderate dorsiflexion and “2” high dorsiflexion (Hardy, 1972), and the lordosis quotient obtained by dividing the total number of lordosis by the number of mounts and intromissions multiplied by 100. Additionally, in the paced mating groups we recorded the return latency to the male compartment after a mount or intromission and the percentage of exits from the male compartment after a mount or intromission.

### 5-Bromo-2′Deoxyuridine Injection

In their first behavioral test females received three injections of the DNA synthesis marker 5-bromo-2′deoxyuridine (BrdU, Figure 1) in 0.9% NaCl (total doses 300 mg/kg). The first injection (100 mg/kg) was administered 1 h before the behavioral test, the second immediately after and the last 1 h after the test.

### Euthanasia

Forty-five days after the BrdU injections, females were sacrificed. They were deeply anesthetized with an overdose of sodium pentobarbital (100 mg/kg), and intracardially perfused with sodium phosphate buffer (PBS, Ph 7.4) followed by 4% paraformaldehyde. Brains were removed and postfixed for 1 h with paraformaldehyde and then cryopreserved in 30% sucrose.

### Immunohistochemistry

**FIGURE 2:**
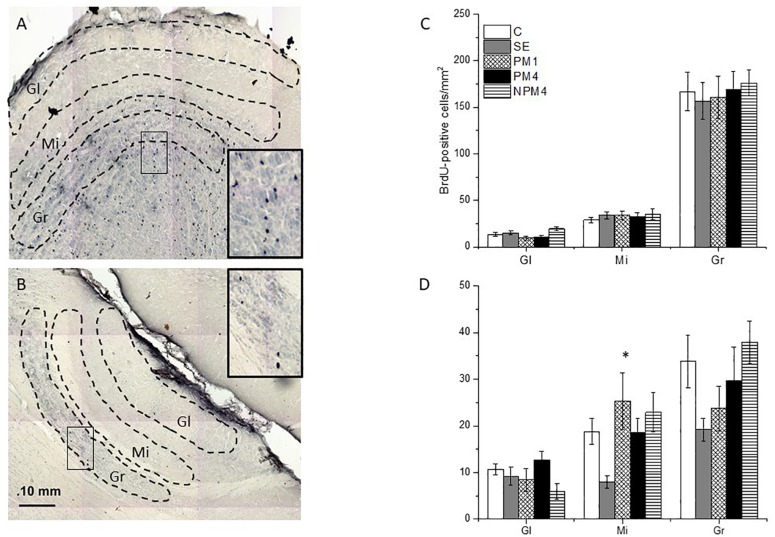
Photomicrographs indicating the areas where BrdU + cells were counted in the glomerular (Gl), mitral (Mi), and granular (Gr) cell layers of the main (MOB; **A**) and accessory (AOB; **B**) olfactory bulb. The number of cells of the different groups is presented for the MOB **(C)** and AOB **(D)**. Female rats were randomly assigned to one of the following groups: (1) Control (C), females that did not receive any sexual stimulation; (2) Social exposure (SE), females exposed in four sessions to a sexually active male without mating; (3) Paced mating one session (PM1), females mated once in pacing conditions; (4) Paced mating four sessions (PM4), females mated in four sessions pacing the sexual interaction; and (5) Non-paced mating four sessions (NPM4), females mated in four sessions without pacing the sexual interaction. Gl layer of MOB: C, PM1, PM4, SE (*n* = 10) NPM4 (*n* = 7); Mi and Gr layer of the MOB: SE (*n* = 10), C and PM4 (*n* = 8) and NPM4 and PM1 (*n* = 7); Gl, Mi, and Gr cell layer of the AOB: SE (*n* = 10) and C, PM1, PM4, and NPM4 (*n* = 8). *Different from SE. *P* < 0.05.

Brains were sliced in the sagittal plane at 30 μm and processed to detect BrdU positive cells (BrdU+, Figures 2A,B). Immunohistochemistry was performed as previously reported (Arzate et al., 2011; Corona et al., 2011, 2016; Unda et al., 2016; Alvarado-Martinez and Paredes, 2018). Brain sections were incubated in HCL 2N at 37°C washed in Tris Buffer (TBS; Tris hydrochloride, Tris Base and Sodium chloride) and incubated with anti-BrdU mouse antibody (BD Bioscience; 1:2000) for 16 h at 4°C. Later, brain sections were incubated in biotinylated anti-mouse IgG (1:500; Vector BA-2000) followed by incubation with the avidin-biotin complex (ABC, Vectastain). To reveal the signal, tissue was incubated in diaminobenzidine. After rinsing brain slices were mounted and coverslipped using permount. As negative controls brain slices without primary antibody and tissue from a rat not injected with BrdU were included. In those tissues no BrdU + cells were visualized.

### Immunofluorescence

To determine if the new cells differentiate into mature neurons, brain slices were incubated in anti BrdU antibody and neuronal nuclei (NeuN) antibody to identify mature neurons. The procedure has been previously reported (Arzate et al., 2013; Corona et al., 2016; de Los Angeles et al., 2016; Unda et al., 2016; Alvarado-Martinez and Paredes, 2018). Briefly, brain sections were incubated with rat anti-BrdU (BD Bioscience, 1:800) and mouse anti-NeuN (1:250, Invitrogen) antibodies for 16 h at 4°C. Later brain slices were incubated in rat anti-IgG Alexa Fluor 488 (1:1100, Invitrogen) and mouse Anti-IgG Alexa Fluor 568 (1:1100, Invitrogen). At the end of the procedure, brain slices were mounted and cover slipped using AquaPoly/Mount (Polysciences, Inc.).

### Quantification of BrdU + and BrdU/NeuN + Cells

To quantify BrdU + cells by immunohistochemistry, photomicrographs were taken using a light Olympus BX60 microscope at 20X amplification using a motorized stage. Three to four brain slices at the level of the AOB and MOB per rat were analyzed (Figures 2A,B, respectively). Brain slices were matched neuroanatomically across all animals. Eight females were evaluated per group. The MOB layers were delimited by three circles of 400 μm in diameter to quantify the number of BrdU + cells. In the AOB three 200 μm diameter circles delimitated the layers. The number of cells in each circle per layer were added and we calculated the mean values from 3 to 4 brain slices. The ImagePro program was used to automatically quantify the number of BrdU + cells in each layer.

For immunofluorescence 20X photomicrographs from four animals in each group were taken of the MOB and AOB using a confocal Zeiss LSM 700. The number of BrdU + cells and the double labeling (BrdU/NeuN+) were evaluated with the orthogonal tool of the Zeiss LSM Image Examiner software.

### Statistical Analysis

Data were analyzed using SigmaPlot 11.0 software; data was not normally distributed (Shapir–Wilk normality test and Equality of variance test). Mount, intromission and ejaculation numbers, and latency, inter intromission interval, lordosis intensity, and lordosis quotient were analyzed with Kruskal Wallis tests. In case of statistically significant differences, a *post hoc* Dunn’s test was used. Return latencies and percentage of exits after a mount and intromission were analyzed by Mann–Whitney *U*-tests. The number of BrdU + and BrdU/NeuN + cells was analyzed with a Kruskal Wallis test, and the percentage of BrdU/NeuN + cells was analyzed by a Ch^2^-test.

## Results

### Sexual Behavior

For each behavioral parameter, the average of the four behavioral tests in the PM4 and NPM4 conditions was obtained. Significant differences were found in the number of intromissions. *Post hoc* tests demonstrated that PM4 females received fewer intromissions than PM1 females. PM1 and PM4 females showed a higher lordosis quotient than NPM4 females. No significant differences between groups were found in the other sexual behavior parameters (Table 1).

**TABLE 1:** Sexual behavior parameters in females that paced the sexual interaction in one (PM1), or four (PM4) sessions and in females that mated in four sessions without pacing the sexual interaction (NPM4).

	PM1	PM4	NPM4
No. Mounts	25 ± 7	10 ± 1	24 ± 7
No. Intromissions	19 ± 3	7 ± 1*	12 ± 2
No. ejaculations	2 ± 0.5	3 ± 0.3	3 ± 0.4
Mount lat.	49 ± 18	40 ± 8	27 ± 7
Intromission lat.	128 ± 60	79 ± 21	110 ± 28
Ejaculation lat.	1182 ± 188	920 ± 121	1162 ± 210
III	128 ± 23	138 ± 18	100 ± 15
Lordosis intensity	1.8 ± 0.1	1.8 ± 0	1.5 ± 0.1
Lordosis quotient	100	100	97 ± 1* +
RL mount	31.4 ± 8	31.2 ± 5.6	
RL intromission	55.7 ± 10.3	67.5 ± 8.6	
PE mount	59.6 ± 9.6	68.7 ± 6.2	

For PM4 and NPM4 the average of the four behavioral tests is reported. Latencies (Lat) are reported in seconds. III inter intromission interval. *Different from PM1. ^+^Different from PM4.

### BrdU Cells

#### MOB

The Kruskal-Wallis test revealed significant differences in the glomerular (*H* = 10.4, 4 df, *P* = 0.034) cell layer of the MOB. However, the *post hoc* Dunn’s test showed no significant differences between groups. As well, no significant difference between groups were found in the number of BrdU + cells that survived in the granular (*H* = 1.1, 4 df, *P* = 0.9) and mitral (*H* = 1.7, 4 df, *P* = 0.786) cell layers of the MOB (Figure 2C).

#### AOB

Significant differences were found in the mitral cell layer (*H* = 14.4, 4 df, *P* = 0.006). *Post hoc* Dunns tests revealed a significant increase of BrdU + cells in the PM1group in comparison to the SE group. No significant differences were found in the number of new cells that survived in the glomerular (*H* = 8.6, 4 df, *P* = 0.07) and granular (*H* = 9.4, 4 df, *P* = 0.052) cell layers of the AOB (Figure 2D).

Our data show that exposure to a sexually active male decreased the number of new cells that survived in the mitral cell layer of the AOB, in comparison to females that mated once pacing the sexual interaction.

### BrdU/NeuN Cells

#### MOB

**FIGURE 3:**
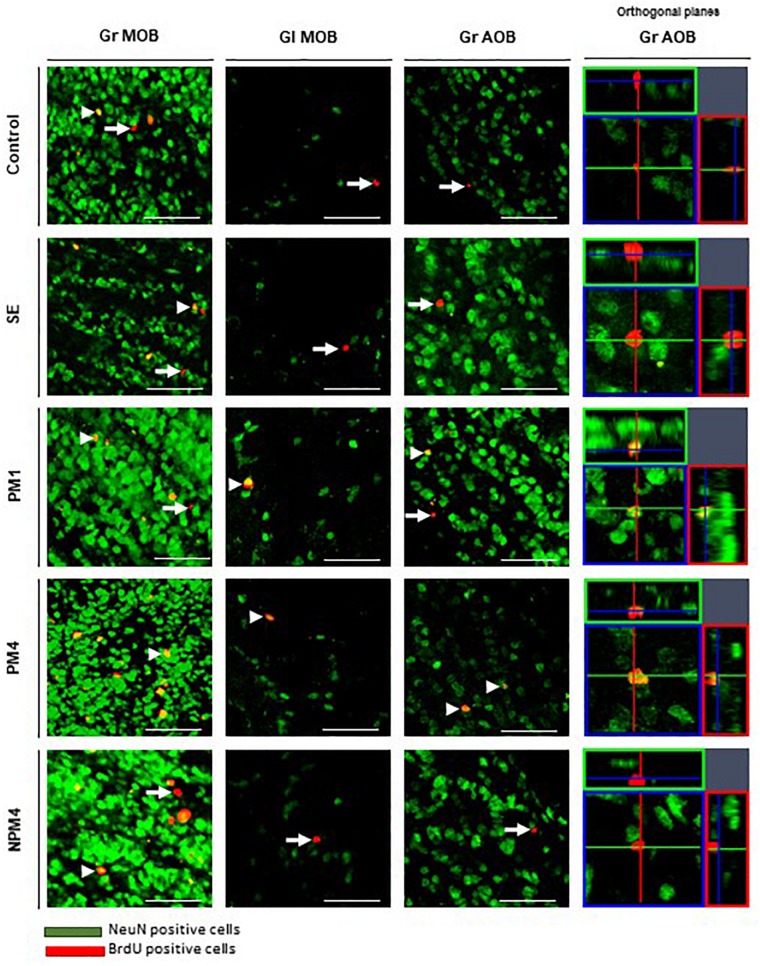
Representative photomicrographs of BrdU/NeuN + cells in the granular (Gr) and glomerular (Gl) layers of the main (MOB) and accessory (AOB) olfactory bulb. Female rats were randomly assigned to one of the following groups: (1) Control (C), females that did not receive any sexual stimulation; (2) Social exposure (SE), females exposed in four sessions to a sexually active male without mating; (3) Paced mating one session (PM1), females mated once in pacing conditions; (4) Paced mating four sessions (PM4), females mated in four sessions pacing the sexual interaction; and (5) Non-paced mating four sessions (NPM4), females mated in four sessions without pacing the sexual interaction. Females were sacrificed 45 days after the first behavioral test. NeuN + cells in green and BrdU + cells in red. Arrows indicate BrdU + cells and arrow heads the BrdU/NeuN + cells, orthogonal planes of Gr AOB cells are showed. Scale bar 50 μm.

Representative photomicrographs of BrdU and BrdU/NeuN + cells are showed in Figure 3. No significant differences were found in the number of BrdU + cells in the granular cell layer (*H* = 7, 4 df, *P* = 0.1). However, PM4 females showed a significant increase in the percentage of new cells that differentiated into neurons in comparison to C, SE, and NPM4 females (Table 2). The glomerular cell layer was not analyzed because we were not able to find a representative number of slices per subject.

**TABLE 2:** Number of BrdU and BrdU/NeuN + cells and percentage of new neurons evaluated by immunofluorescence in the granular (Gr) cell layer of the main olfactory bulb (MOB) and Gr and glomerular (Gl) cell layer of the accessory olfactory bulb (AOB) in the different groups.

	C	SE	PM1	PM4	NPM4
**MOB Gr**					
BrdU	70 ± 9	83 ± 11	95 ± 20	117 ± 20	128 ± 22
BrdU/NeuN	52 ± 5	64 ± 12	81 ± 18	102 ± 16	95 ± 19
Percentage	75	75	85	88*	74
**AOB Gl**					
BrdU	1.6 ± 1	4.1 ± 1	5.3 ± 2	2.4	6.9 ± 1
BrdU/NeuN	1.2 ± 1	1 ± 1	3.7 ± 2	2 ± 1	1.6 ± 1
Percentage	38	17^+^	66*	75*	23^+^
**AOB Gr**					
BrdU	5.7 ± 3	8.5 ± 2	8.5 ± 1	11.4 ± 2	10.2 ± 1
BrdU/NeuN	3.7 ± 2	4.9 ± 1	5.3 ± 1	7.3	5 ± 1
Percentage	58	60	62	75*	38

*Different from C, SE, and NPM4. ^+^Different from C.

#### AOB

No significant differences were found in the number of BrdU cells in the granular and glomerular cell layers (*H* = 3.8, 4 df, *P* = 0.4; *H* = 8.4, 4 df, *P* = 0.1, respectively). In the glomerular cell layer, PM4 and PM1 females showed an increase in the percentage of new neurons, whereas SE and NPM4 females showed a decrease in comparison to C females. In the granular cell layer, the PM4 group had a higher percentage of new neurons in comparison to the C, SE, and NPM4 groups (Table 2).

## Discussion

**FIGURE 4:**
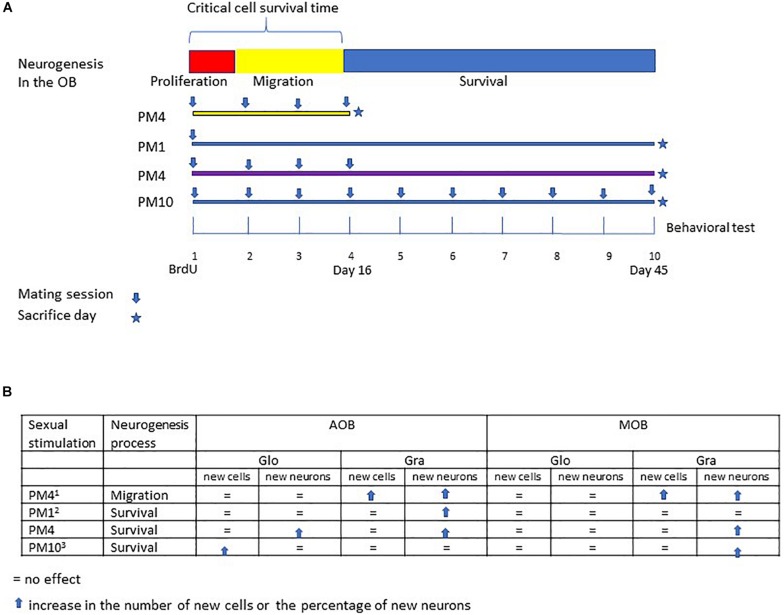
Summary of the different studies evaluating the effects of repeated sexual stimulation in female rats that mated pacing the sexual interaction upon cell migration and survival in the glomerular and granular cell layers of the main and accessory olfactory bulb. **(A)** schematic representation of the neurogenesis process; cell proliferation (48 h), migration (15 days) and survival (45 days), and experimental design of previous studies evaluating the effects of mating sessions over cell migration (PM4), survival of cells after one paced mating session (PM1), and continues sexual stimulation during migration and cell survival (PM10). In the present study females mated four times in pacing conditions during the critical cell survival time (first 16 days) and were sacrificed 45 days after the first paced mating session. **(B)** Summary of the principal findings in the present and previous studies (^1^Arzate et al., 2013; ^2^Corona et al., 2016; ^3^Alvarado-Martinez and Paredes, 2018).

Sexual behavior is a physiological stimulation that modulates adult neurogenesis in the olfactory bulbs and dentate gyrus of the hippocampus, review in Bedos et al. (2018) and Portillo et al. (2019). Sexual behavior in mammals is innate, however, experience shapes this behavior (Herrera-Morales et al., 2019). For example, sexually experienced female rats show a higher preference for a male (Woodson et al., 2002), favoring discrimination between sexual and non-sexual partners (Nofrey et al., 2008). As well, sexual experience increases neuronal activity in the amygdala, medial preoptic area and nucleus accumbens (Hosokawa and Chiba, 2007), brain areas important in the control of sexual behavior. Repeated sexual interaction also potentiates cell migration in the olfactory bulbs. Female rats that mated one or four times pacing the sexual interaction showed a higher percentage of new cells that migrate in the granular cell layer of the AOB. On the other hand, only females that mated in pacing conditions in four sessions showed more new cells in the mitral cell layer of the AOB and granular layer of the MOB. Repeated sexual experience in paced mating conditions also influences the differentiation lineage of new cells. Females that mated one or four times in pacing conditions showed more neurons in the granular cell layer of the AOB. Only those females that mated four times showed an increase in neurons in the mitral and granular cell layer of the MOB 16 days after the behavioral test (Arzate et al., 2013). In the present study, we evaluated if repeated sexual stimulation during the first 16 days after the first sexual experience, favors the survival of new cells in the olfactory bulbs and increases their neuronal differentiation 45 days after mating (Figure 4). During repeatedly mating several factors can be release and those can influence cell survival.

Our results show that female rats that mated, once a week for 4 weeks, pacing the sexual interaction, (PM4) showed an increase in the percentage of new cells that differentiated into neurons (45 days after the first sexual experience) in the granular cell layer of the MOB and AOB in comparison to females that mated four times but did not pace the sexual interaction (NPM4), females exposed to a sexually active male (SE) and control (C) females. Similarly, an increase in the percentage of new neurons that survived was found in the glomerular cell layer of the AOB in PM1 and PM4 females. These results agree with previous studies demonstrating that in female rats the first mating experience in pacing conditions increases the percentage of new cells that survive and differentiate into neurons in the (Corona et al., 2016). Additionally, when the number of paced mating sessions increased, the female rats that mated 10 times (once per week) showed an increase in the percentage of new neurons in the MOB but not in AOB (Alvarado-Martinez and Paredes, 2018). Thus, sexual activity during the first 16 days, a critical period for cell survival enhances the neuronal phenotype in the AOB but continuous sexual stimulation after this critical time appears to inhibit it.

In female rats, exposure to male sexual cues without mating increases cell proliferation. Exposing females for 1 h to a male or non-sexually related odor (banana scent) increases proliferation in the SVZ. Male pheromone induced cell proliferation has been reported in mice, voles and ungulates. Female mice exposed to male pheromones (7–21 days) for long periods show an increase in cell proliferation in the SVZ and hippocampus (Larsen et al., 2008; Koyama et al., 2013, 2014; Hoffman et al., 2015). In prairie voles, male pheromones increases cell proliferation and differentiation into immature neurons in the RMS (Smith et al., 2001). In ungulates exposure to males for 48 h increases the number of new cells that proliferate in the dentate gyrus of the hippocampus (Hawken et al., 2009). However exposure to a male once for 1 h does not induce migration or survival into the olfactory bulbs in female rats (Arzate et al., 2013; Corona et al., 2016; Alvarado-Martinez and Paredes, 2018). In female mice chronic exposure to male pheromones (up to 21 days) increases cell survival (Larsen et al., 2008; Nunez-Parra et al., 2011; Oboti et al., 2011; Wu et al., 2013). In the present study, we evaluated if continuous exposure to a male (1 h once a week for 4 weeks) increases survival of the new cells to the olfactory bulbs. We found that this olfactory stimulation was not enough to increase survival of the new cells. Maybe, as occurs in mice, the exposure to a male or his pheromones needs to be chronic (daily for 3 weeks) to increase cell survival. Only future studies can answer this question.

Previous studies from our research group demonstrated that in female rats repeated mating (four sexual behavioral tests) potentiates the increase in the number of new cells that reach the granular cell layer of the AOB and MOB (Arzate et al., 2013) in comparison to one paced mating session. In the present study, we evaluated if repeated paced mating increases the number of new cells that survive in the olfactory bulbs. Although repeated sexual stimulation favors the differentiation of new cells into neurons in the olfactory bulb, this behavior does not increase the number of new cells that survive. Females that mated once, pacing the sexual interaction showed an increase in the number of new cells that survived in the mitral cell layer of the AOB in comparison to females exposed to a male. Around 95% of the new cells differentiate into granular neurons and a few into periglomerular neurons which are integrated into the glomerular cell layer (Petreanu and Alvarez-Buylla, 2002; Bagley et al., 2007; Whitman and Greer, 2009). There are no reports indicating that the new cells integrate into the mitral cell layer of the olfactory bulbs. Since this cell population dose not express NeuN (Mullen et al., 1992) we were unable to determine if they are neurons, suggesting that these new cells differentiate into glia. Glial cells can modulate neuronal activity of the mitral neurons, which project to several cortical and subcortical targets. Moreover, mitral cell activity plays a fundamental role in sexual receptivity and mate recognition because their inhibition in the AOB decrease the lordosis response in female rodents (McCarthy et al., 2017). Mitral cells respond specifically to their male sexual partner pheromones, a process important in mate recognition (Brennan et al., 1990; Brennan and Binns, 2005). Further studies are needed to determine if the new cells in the mitral cell layer are neurons or glial cells and their possible relevance in sexual receptivity and mate recognition. Thus, mating four times in pacing or non-pacing conditions did not increase the number of new cells that survive in the AOB or MOB. Similarly, we demonstrated that the first sexual experience in pacing and non-pacing conditions or exposure to a male did not increase the number of new cells that survived in the granular and glomerular cell layer of the AOB and MOB (Corona et al., 2016). In another study we demonstrated that paced mating once a week for 10 consecutive weeks did not increase the number of new cells that survived in the AOB or MOB 45 days after the first sexual test (Alvarado-Martinez and Paredes, 2018). A summary of the different changes induced by mating is presented in Figure 4. What is clear is that repeated mating does not potentiate cell survival, but it does potentiate neurogenesis. Similar results were reported in the hippocampus, Leuner and coworkers (Leuner et al., 2010) demonstrated in male rats that one sexual experience or mating for 14 days induces a similar increase in the number of new cells that survive in the dentate gyrus of the hippocampus. Taken together, our results indicate that mating in paced and no-paced conditions increases cell proliferation in the RMS (Corona et al., 2016). In Alvarado-Martinez and Paredes (2018) and the present study, female rats always mated with the same male to receive similar sexual stimulation and avoid exposure to different male pheromones. Mating with different sexual partners has negative effects on neurogenesis. Male rats that mate four times with different females show decreased neurogenesis in the dentate gyrus of the hippocampus in comparison to males that mate with the same female several times (Spritzer et al., 2016). Furthermore, males display more mounts and intromission when they mate with the same female than when they mate with different sexual partners (Spritzer et al., 2016). However, it has been demonstrated that females are able to recognize the male with whom they mate, and the pheromones from a previous partner do not induce neuronal activation (Brennan et al., 1990; Xu et al., 2016; Gao et al., 2017). In female sheep, exposure to a non-familiar male increases luteinizing hormone pulse frequency; this neuroendocrine response is not observed when females are exposed to a familiar male (Hawken et al., 2009). Taken together the results indicate that one sexual stimulation increases the number of cells that reach the OB 15 days after paced mating. Repeated paced mating increases the number of cells in the same period. However, one or four sessions of paced mating increases the survival of new neurons in the olfactory bulbs 45 days after the sexual behavior test.

## Data Availability Statement

The datasets generated for this study are available on request to the corresponding author.

## Ethics Statement

The animal study was reviewed and approved by the Bioethics Committee of the Instituto de Neurobiología, and were carried out in accordance with the “Reglamento de la Ley General de Salud en Materia de Investigación para la Salud” of the Mexican Health Ministry, and NIH guidelines.

## Author Contributions

WP supervised research, contributed with funding, and wrote the manuscript. GO performed the experiments, analyzed data, and prepared the figures. RP planned the experiments, supervised research, contributed with funding, and wrote the manuscript.

## Conflict of Interest

The authors declare that the research was conducted in the absence of any commercial or financial relationships that could be construed as a potential conflict of interest.

The reviewer PP declared a past co-authorship with one of the authors RP with the authors to the handling Editor.

## References

[B1] Agmo A. Berenfeld R. (1990). Reinforcing properties of ejaculation in the male rat: role of opioids and dopamine. *Behav. Neurosci.* 104 177–182. 10.1037/0735-7044.104.1.177 2156520

[B2] Alvarado-Martinez R. Paredes R. G. (2018). Incorporation of new neurons in the olfactory bulb after paced mating in the female rat. *Behav. Brain Res.* 343 95–101. 10.1016/j.bbr.2018.02.006 29425917

[B3] Arzate D. M. Portillo W. Corona R. Paredes R. G. (2013). Repeated paced mating promotes the arrival of more newborn neurons in the main and accessory olfactory bulbs of adult female rats. *Neuroscience* 232 151–160. 10.1016/j.neuroscience.2012.12.014 23262235

[B4] Arzate D. M. Portillo W. Rodriguez C. Corona R. Paredes R. G. (2011). Extended paced mating tests induces conditioned place preference without affecting sexual arousal. *Horm. Behav.* 59 674–680. 10.1016/j.yhbeh.2010.08.016 20816964

[B5] Bagley J. LaRocca G. Jimenez D. A. Urban N. N. (2007). Adult neurogenesis and specific replacement of interneuron subtypes in the mouse main olfactory bulb. *BMC Neurosci.* 8:92. 10.1186/1471-2202-8-92 17996088 PMC2238759

[B6] Bedos M. Portillo W. Paredes R. G. (2018). Neurogenesis and sexual behavior. *Front. Neuroendocrinol.* 51 68–79. 10.1016/j.yfrne.2018.02.004 29438737

[B7] Brennan P. Kaba H. Keverne E. B. (1990). Olfactory recognition: a simple memory system. *Science* 250 1223–1226. 10.1126/science.2147078 2147078

[B8] Brennan P. A. Binns E. K. (2005). Vomeronasal mechanisms of mate recognition in mice. *Chem. Senses* 30 (Suppl. 1), i148–i149. 10.1093/chemse/bjh157 15738084

[B9] Coria-Avila G. A. Gavrila A. M. Boulard B. Charron N. Stanley G. Pfaus J. G. (2008). Neurochemical basis of conditioned partner preference in the female rat: II. Disruption by flupenthixol. *Behav. Neurosci.* 122 396–406. 10.1037/0735-7044.122.2.396 18410178

[B10] Coria-Avila G. A. Jones S. L. Solomon C. E. Gavrila A. M. Jordan G. J. Pfaus J. G. (2006). Conditioned partner preference in female rats for strain of male. *Physiol. Behav.* 88 529–537. 10.1016/j.physbeh.2006.05.001 16757008

[B11] Corona R. Larriva-Sahd J. Paredes R. G. (2011). Paced-mating increases the number of adult new born cells in the internal cellular (granular) layer of the accessory olfactory bulb. *PLoS One* 6:e19380. 10.1371/journal.pone.0019380 21637743 PMC3103495

[B12] Corona R. Retana-Marquez S. Portillo W. Paredes R. G. (2016). Sexual behavior increases cell proliferation in the rostral migratory stream and promotes the differentiation of the new cells into neurons in the accessory olfactory bulb of female rats. *Front. Neurosci.* 10:48. 10.3389/fnins.2016.00048 26955325 PMC4767934

[B13] de Los Angeles G. A. Del Carmen R. O. Wendy P. M. Socorro R. M. (2016). Tactile stimulation effects on hippocampal neurogenesis and spatial learning and memory in prenatally stressed rats. *Brain Res. Bull.* 124 1–11. 10.1016/j.brainresbull.2016.03.003 26993794

[B14] Erskine M. S. (1989). Solicitation behavior in the estrous female rat: a review. *Horm. Behav.* 23 473–502. 10.1016/0018-506x(89)90037-8 2691387

[B15] Gao Y. Budlong C. Durlacher E. Davison I. G. (2017). Neural mechanisms of social learning in the female mouse. *eLife* 6:e25421. 10.7554/eLife.25421 28621665 PMC5531829

[B16] Hardy D. F. (1972). Sexual behavior in continuously cycling rats. *Behaviour* 41 288–297. 10.1163/156853972x00068 5063409

[B17] Hawken P. A. Jorre T. J. Rodger J. Esmaili T. Blache D. Martin G. B. (2009). Rapid induction of cell proliferation in the adult female ungulate brain (*Ovis aries*) associated with activation of the reproductive axis by exposure to unfamiliar males. *Biol. Reprod.* 80 1146–1151. 10.1095/biolreprod.108.075341 19176880

[B18] Herrera-Morales W. V. Herrera-Solis A. Nunez-Jaramillo L. (2019). Sexual behavior and synaptic plasticity. *Arch. Sex. Behav.* 48 2617–2631. 10.1007/s10508-019-01483-2 31270644

[B19] Hoffman E. Pickavance L. Thippeswamy T. Beynon R. J. Hurst J. L. (2015). The male sex pheromone darcin stimulates hippocampal neurogenesis and cell proliferation in the subventricular zone in female mice. *Front. Behav. Neurosci.* 9:106. 10.3389/fnbeh.2015.00106 25972792 PMC4413791

[B20] Hosokawa N. Chiba A. (2007). Effects of sexual experience on conspecific odor preference and male odor-induced activation of the vomeronasal projection pathway and the nucleus accumbens in female rats. *Brain Res.* 1175 66–75. 10.1016/j.brainres.2007.07.071 17870062

[B21] Koyama S. Soini H. A. Foley J. Novotny M. V. Lai C. (2013). Stimulation of cell proliferation in the subventricular zone by synthetic murine pheromones. *Front. Behav. Neurosci.* 7:101. 10.3389/fnbeh.2013.00101 23964214 PMC3734356

[B22] Koyama S. Soini H. A. Foley J. Novotny M. V. Lai C. (2014). Pheromone-induced cell proliferation in the murine subventricular zone. *Biochem. Soc. Trans.* 42 882–885. 10.1042/BST20140112 25109973

[B23] Kudwa A. E. Dominguez-Salazar E. Cabrera D. M. Sibley D. R. Rissman E. F. (2005). Dopamine D5 receptor modulates male and female sexual behavior in mice. *Psychopharmacology* 180 206–214. 10.1007/s00213-005-2150-5 15696326

[B24] Larsen C. M. Kokay I. C. Grattan D. R. (2008). Male pheromones initiate prolactin-induced neurogenesis and advance maternal behavior in female mice. *Horm. Behav.* 53 509–517. 10.1016/j.yhbeh.2007.11.020 18258236

[B25] Leuner B. Glasper E. R. Gould E. (2010). Sexual experience promotes adult neurogenesis in the hippocampus despite an initial elevation in stress hormones. *PLoS One* 5:e11597. 10.1371/journal.pone.0011597 20644737 PMC2904381

[B26] Martinez I. Paredes R. G. (2001). Only self-paced mating is rewarding in rats of both sexes. *Horm. Behav.* 40 510–517. 10.1006/hbeh.2001.1712 11716580

[B27] McCarthy E. A. Kunkhyen T. Korzan W. J. Naik A. Maqsudlu A. Cherry J. A. (2017). A comparison of the effects of male pheromone priming and optogenetic inhibition of accessory olfactory bulb forebrain inputs on the sexual behavior of estrous female mice. *Horm. Behav.* 89 104–112. 10.1016/j.yhbeh.2016.12.011 28065711 PMC5359026

[B28] McClintock M. K. Adler N. T. (1978). Induction of persistent estrus by airborne chemical communication among female rats. *Horm. Behav.* 11 414–418. 10.1016/0018-506x(78)90041-7 572810

[B29] Mullen R. J. Buck C. R. Smith A. M. (1992). NeuN, a neuronal specific nuclear protein in vertebrates. *Development* 116 201–211. 1483388 10.1242/dev.116.1.201

[B30] Nofrey B. Rocha B. Lopez H. H. Ettenberg A. (2008). The effects of sexual experience and estrus on male-seeking motivated behavior in the female rat. *Physiol. Behav.* 95 533–538. 10.1016/j.physbeh.2008.08.002 18761024 PMC2574631

[B31] Nunez-Parra A. Pugh V. Araneda R. C. (2011). Regulation of adult neurogenesis by behavior and age in the accessory olfactory bulb. *Mol. Cell. Neurosci.* 47 274–285. 10.1016/j.mcn.2011.05.003 21600286 PMC3137699

[B32] Oboti L. Schellino R. Giachino C. Chamero P. Pyrski M. Leinders-Zufall T. (2011). Newborn interneurons in the accessory olfactory bulb promote mate recognition in female mice. *Front. Neurosci.* 5:113. 10.3389/fnins.2011.00113 21994486 PMC3182443

[B33] Parada M. Vargas E. B. Kyres M. Burnside K. Pfaus J. G. (2012). The role of ovarian hormones in sexual reward states of the female rat. *Horm. Behav.* 62 442–447. 10.1016/j.yhbeh.2012.07.012 22902894

[B34] Peretto P. Paredes R. G. (2014). “Social cues, adult neurogenesis, and reproductive behavior,” in *Neurobiology of Chemical Communication*, ed. Mucignat-Caretta C. (Boca Raton, FL: CRC Press). 24830028

[B35] Petreanu L. Alvarez-Buylla A. (2002). Maturation and death of adult-born olfactory bulb granule neurons: role of olfaction. *J. Neurosci.* 22 6106–6113. 10.1523/jneurosci.22-14-06106.2002 12122071 PMC6757952

[B36] Pfaus J. G. Kippin T. E. Coria-Avila G. A. Gelez H. Afonso V. M. Ismail N. (2012). Who, what, where, when (and maybe even why)? How the experience of sexual reward connects sexual desire, preference, and performance. *Arch. Sex. Behav.* 41 31–62. 10.1007/s10508-012-9935-5 22402996

[B37] Portillo W. Bedos M. Nuñez M. Paredes R. (2018). “Neurobiology of reproductive behavior, the role of neurogenesis,” in *Encyclopedia of Animal Behavior*, ed. Choe J. (New York, NY: Elsevier), 1–9.

[B38] Portillo W. Bedos M. Nuñez M. Paredes R. (2019). “Neurobiology of reproductive behavior, the role of neurogenesis,” in *Encyclopedia of Animal Behavior*, 2 Edn, ed. Jae C. (Cambridge, MA: Academic Press), 3048.

[B39] Smith M. T. Pencea V. Wang Z. Luskin M. B. Insel T. R. (2001). Increased number of BrdU-labeled neurons in the rostral migratory stream of the estrous prairie vole. *Horm. Behav.* 39 11–21. 10.1006/hbeh.2000.1630 11161879

[B40] Spritzer M. D. Curtis M. G. DeLoach J. P. Maher J. Shulman L. M. (2016). Sexual interactions with unfamiliar females reduce hippocampal neurogenesis among adult male rats. *Neuroscience* 318 143–156. 10.1016/j.neuroscience.2016.01.015 26794592 PMC4753127

[B41] Ulloa M. Portillo W. Diaz N. F. Young L. J. Camacho F. J. Rodriguez V. M. (2018). Mating and social exposure induces an opioid-dependent conditioned place preference in male but not in female prairie voles (*Microtus ochrogaster*). *Horm. Behav.* 97 47–55. 10.1016/j.yhbeh.2017.10.015 29111331 PMC5803795

[B42] Unda N. M. Portillo W. Corona R. Paredes R. G. (2016). Sexual stimulation increases the survival of new cells in the accessory olfactory bulb of the male rat. *Front. Neurosci.* 10:65. 10.3389/fnins.2016.00065 26973447 PMC4771754

[B43] Whitman M. C. Greer C. A. (2009). Adult neurogenesis and the olfactory system. *Prog. Neurobiol.* 89 162–175. 10.1016/j.pneurobio.2009.07.003 19615423 PMC2748178

[B44] Winner B. Cooper-Kuhn C. M. Aigner R. Winkler J. Kuhn H. G. (2002). Long-term survival and cell death of newly generated neurons in the adult rat olfactory bulb. *Eur. J. Neurosci.* 16 1681–1689. 10.1046/j.1460-9568.2002.02238.x 12431220

[B45] Woodson J. C. Balleine B. W. Gorski R. A. (2002). Sexual experience interacts with steroid exposure to shape the partner preferences of rats. *Horm. Behav.* 42 148–157. 10.1006/hbeh.2002.1816 12367568

[B46] Wu J. H. Han Y. T. Yu J. Y. Wang T. W. (2013). Pheromones from males of different familiarity exert divergent effects on adult neurogenesis in the female accessory olfactory bulb. *Dev. Neurobiol.* 73 632–645. 10.1002/dneu.22090 23696538

[B47] Xu P. S. Lee D. Holy T. E. (2016). Experience-dependent plasticity drives individual differences in pheromone-sensing neurons. *Neuron* 91 878–892. 10.1016/j.neuron.2016.07.034 27537487 PMC5003430

